# Individual and community-level factors associated with caesarean section in Haiti: secondary analysis of data from the 2016–2017 Haitian Demographic and Health Survey

**DOI:** 10.1186/s41182-023-00513-z

**Published:** 2023-04-17

**Authors:** David Jean Simon, Stanley Jean-Baptiste, Roodjmie Nazaire, Ghislaine Joseph, Joseph Arcelin Carmil, Fanor Joseph, Vénunyé Claude Kondo Tokpovi

**Affiliations:** 1 Bureau d’Etudes et de Recherche en Statistiques Appliquées, Suivi et Evaluation (BERSA-SE), Port-au-Prince, Haiti; 2 Universidad de Santiago de Chile, Santiago, Chile; 3 Université d’Etat d’Haïti (UEH), Faculté de Médecine et de Pharmacie (FMP), Port-au-Prince, Haiti; 4 Centre de Recherche Cultures Arts Sociétés (CELAT), University of Laval, Quebec City, Canada; 5 Université de Bordeaux, UMR Passages, Pessac, France; 6 University of Antananarivo, Doctoral School of Social and Human Sciences, Antananarivo, Madagascar; 7 Groupe de Recherche sur l’inadaptation Psychosociale (GRIP), University of Laval, Quebec City, Canada

**Keywords:** Caesarean section, Prevalence, Delivery, Demographic and Health Survey, Factors, Haiti

## Abstract

**Introduction:**

For several decades, the rate of caesarean section (CS) has been increasing in the world. In some countries, the CS rate is below the WHO recommended range (10–15%), while in other countries, it is significantly higher. The aim of this paper was to identify individual and community-level factors associated with CS in Haiti.

**Methods:**

Secondary data analysis was conducted on nationally representative cross-sectional survey data from the 2016–2017 Haitian Demographic and Health Survey (HDHS). The analysis was restricted to 6303 children born in 5 years prior the survey (of the interviewed women). The study population’ characteristics, and the prevalence of CS were analysed using descriptive analysis (univariate/bivariate). In addition, multilevel binary logistic regression analysis was performed to identify factors associated with CS. Both descriptive and multivariate analysis were conducted using STATA 16.0 software (Stata Corp, Tex, USA). Statistical significance was declared at *p* < 0.05.

**Results:**

The overall prevalence of CS delivery was estimated at 5.4% (95% CI 4.8–6.0) in Haiti. Results also revealed that mothers aged 35 and above (aOR = 1.38; 95% CI 1.00–1.96); who attended secondary (aOR = 1.95; 95% CI 1.39–2.76) and higher education level (aOR = 3.25; 95% CI 1.92–5.49); who were covered by health insurance (aOR = 2.57; 95% CI 1.57–4.19); with less than 3 children (aOR = 4.13; 95% CI 2.18–7.85) or 3–4 children (aOR = 2.07; 95% CI 1.09–3.94); who received 9 or more antenatal visits (aOR = 2.21; 95% CI 1.40–3.50) were significantly more likely to deliver by CS. Children in communities with high preponderance of private health facilities had greater odds to be delivered through CS (aOR = 1.90; 95% CI 1.25–2.85). Furthermore, children with an average birth weight (aOR = 0.66; 95% CI 0.48–0.91) were less likely to be delivered through CS than their counterparts with high birth weight.

**Conclusions:**

While the CS prevalence was low in Haiti, it masks significant geographic, social and economic disparities. To better develop and implement maternal and child health programs that address CS deliveries, the government authorities and NGOs operating in the field of women’s health in Haiti should take these disparities into account.

## Introduction

Defined as “a fetal delivery through an open abdominal incision (laparotomy) and an incision in the uterus (hysterotomy)” [1], the caesarean section (CS), C-section, or caesarean birth, has significantly increased over the past 3 decades worldwide in both developing and developed countries, and is even considered a global public health issue. In 1990, the CS rate was estimated at about 7% compared to over 21% in 2021 [2]. Although the CS rate has tripled during this period, according to WHO, population-based CS rates of greater than 10% are not associated with reductions in maternal and newborn mortality rates [3, 4]. In addition, below 10%, the need for CS is not fully met, which can result in excess maternal and perinatal mortality [5]. WHO has recommended the ideal rate for CS deliveries to be between 10 and 15% [4].

There are great disparities in a woman’s access to caesarean sections [5–9]. Based on the latest UN estimates, the CS rate is 9.2% in Africa, 23.1% in Asia, 25.7% in Europe, 42.8% in Latin America and the Caribbean, 31.6% in Northern America, and 21.4% in Oceania [2]. There are also large intra-regional disparities [4]. For instance, in Latin America and the Caribbean, the magnitude of CS range from around 5% in Haiti [5] to 58% in Dominican Republic [10]. In Niger, Madagascar, Chad, and Mali, the CS rates are less than 5%, while it is 19.1% in Algeria and 16% in Ghana and Morocco [7, 11–13]. Moreover, in Cyprus, Georgia, Romania and Italy, CS rates are 35% or higher compared to around 20% in France and Denmark [14].

Caesarean sections have many benefits [15]. When a vaginal delivery is risky, it is safer for both mothers and babies [16, 17]. A recent study has also found that women who have CS have a lower risk of urinary incontinence and pelvic prolapse [15]. However, when it is unplanned and performed under inappropriate conditions, it can have adverse effects on the health of mothers and newborns, as well as on future pregnancies [15]. These might include: infection, hemorrhage, blood clots, injury to the bowel or bladder, abnormalities of the placenta in future pregnancies, and obstetric fistula [18, 19]. As for the baby, it may have trouble breastfeeding and may be at greater risk for breathing problems [3, 20, 21]. Besides, evidences showed that having a CS increases the risk of complications in a later pregnancy and in other surgeries [22, 23].

Several studies have indicated that individual and community-level factors, such as: mother’s age [21, 24, 25], place of residence, region, education level [8, 24, 26, 27], wealth and occupational status [28–30], exposure to mass media [31], religion [24, 32], antenatal visits [8, 29, 33], age at first marriage, birth order, hypertension, obesity [29, 34], type of health facility, size of child at birth, birth complications [6, 35, 36], being covered by health insurance and number of living children [37–39] are the main factors associated with CS deliveries. Similarly, other studies suggest that psychological factors which may be due to fear related to prolonged labor and vaginal delivery pain increase the likelihood of undergoing CS [40, 41].

Haiti has one of the lowest CS rates in the world, although it has been increasing in recent decades (1.6% in 1995 compared to about 5% in 2020) [2, 5, 10]. The country is also characterized by high maternal mortality ratio (529 maternal deaths per 100,000 live births) [42] due, partly, to mostly preventable complications in pregnancy and delivery [43]. Recent reports have indicated that some maternal deaths could be averted by CS delivery and thus Haitian women who have obstetric complications should undergo CS [44, 45]. Given that complications occur in about 40% of pregnancies in Haiti [46], understanding the enablers and barriers to CS is an important first step toward improving maternal and child health services and addressing health inequalities [2]. This study, therefore, aimed to identify individual and community-level factors associated with CS in Haiti.

## Materials and methods

### Study setting

Haiti is a Caribbean country with 27,750 km^2^ of coverage and the most populous country in this region with approximately 11,7 million people in 2021 [47]. Administratively, the Republic of Haiti is divided into 10 departments (Ouest, Sud, Sud-Est, Grand’Anse, Nippes, Nord, Nord-Ouest, Nord-Est, Centre, and Artibonite), 41 districts, 140 municipalities and 570 communal sections [42]. Ouest, subdivided into Aire Métropolitaine de Port-au-Prince and Reste-Ouest, contains over 35% of the Haitian population [48]. Slightly more than 60% of its population are under 30 years [49] and about 25% live below the national extreme poverty line ($1,23 per day) [50] with a life expectancy at birth of 66,1 years for women and 60,4 years for men [47].

### Data source and sample

The study used the Child data set (KR) from the current Demographic and Health Survey (DHS) conducted in Haiti between November 2016 and April 2017 [42]. The 2016–2017 HDHS is a nationally representative survey data set conducted and collected as a collaboration between Haitian Institute for Children, the Haitian Bureau of Statistics and the Ministry of Public Health and Population, with technical support from ICF through the DHS Program of the United States Agency for International Development (USAID). The survey was designed to capture information on population socio-demographics, maternal and child health, and a variety of health indicators including caesarean section, for the country as a whole, for urban and rural areas separately, and for each of the 10 departments in Haiti [42].

A two-stage stratified sampling design was applied that involved randomly selecting the sampling clusters that were created in the first stage, followed by randomly selecting households per cluster with equal probabilities in a systematic approach in the second stage. Four questionnaires were used for the data collection: Household Questionnaire, Women’s Questionnaire, Men’s Questionnaire and Biomarker Questionnaire. Detailed information regarding the HDHS sampling and data collection have been published elsewhere [42]. Out of 13,405 households interviewed, 14,371 women of childbearing age (15–49 years) were successfully interviewed, with a response rate of 98.9% [42]. Our study focused on children born in 5 years prior the survey (of the interviewed women) (*n* = 6303).

### Study variables

#### Dependent variable

The outcome variable was CS delivery. To derive this variable, interviewed mothers were asked whether their last-born in 5 years prior the survey was delivered by CS. The responses were dichotomized and classified originally as "No (0)" or "Yes (1)".

#### Independent variables

These include socio-demographic, maternal, and children characteristics, such as mother’s age ("less than 25 years", "25–34", "35 and above"), place of residence ("urban", "rural"), region ("Aire Métropolitaine de Port-au-Prince", "Reste-Ouest", "Sud-Est", "Nord", "Nord-Est", "Artibonite", "Centre", "Sud", "Grand’Anse/Nippes", "Nord’Ouest"), religion ("Christian", "Non-Christian"), mother’s education level ("primary or no formal education", "secondary", and "higher"), currently working ("yes", "no"), being covered by health insurance ("yes", "no"), number of living children ("less than 3", "3–4", "5 and above"), number of antenatal visits ("less than 4", "4–8", "9 and above", "don’t know/missing"), type of facility ("public", "private", "home/others"), baby’s birth weight ("average", "low birth weight", "big baby"), and wealth index ("poorest", "poorer", "middle", "richer", "richest"). The household wealth index was a composite score measured by ownership of household items and facilities based on a DHS-generated quintile index. Detailed information about the wealth index construction can be found in the DHS guide [51]. Explanatory variables were chosen based on prior evidence as well as their availability in the Child data set (KR) [8, 29, 32, 39, 52–57].

### *Statistical *analysis

This study employed both descriptive and multivariate analysis. Univariate analysis illustrated frequencies and percentages to describe the study population’s profile. Furthermore, cross-tabulations of each independent variable and CS were applied to estimate the prevalence of CS in Haiti and for inferential analysis. A chi-squared test ascertained whether there was any association between population characteristics and the outcome variable using *p* value < 0.05 as cut of points. Variables with *p* value > 0.20 were excluded from the multivariate analysis. Multilevel analysis (a two-level mixed-effects logistic regression model) was performed to identify significant factors associated with CS births, since the 2016–2017 HDHS data are hierarchical (individual “level 1” variables were nested within community “level 2” variables) [42]. While doing the analysis, we have fitted four models: null model (Model-0), model 1 (Model-I), model 2 (Model-II), and model 3 (Model-III). The null model was fitted with only the outcome variable [58]. Model 1, model 2, and model 3 were fitted using individual-level variables, community-level variables, and both individual and community-level variables, respectively. Results of fixed effects were reported as adjusted odds ratios (aOR) with their corresponding 95% confidence intervals (CI). The random effect was interpreted using the Intra-class Correlation Coefficient (ICC) and the Proportional Change in Variance (PCV) and compared across the progressive models by looking at them. Moreover, the variance inflation factor (VIF) was used to assess multi-collinearity. None of the variables displayed multi-collinearity problems (all VIF < 10, Mean VIF = 2.12) [59]. Log-likelihood and Akaike Information Criterion (AIC) were used to verify model fitness, and a model with the highest log-likelihood and lowest AIC has been deemed as a best-fit model [60]. All analyses were weighted to get unbiased estimates, and carried out in STATA 16.0 software (Stata Corp, Tex, USA) using "*svy*" command to adjust for the complex sampling structure of the data. Statistical significance was declared at *p* < 0.05.

### Ethical consideration

The 2016–2017 HDHS survey obtained ethical clearance from the Ethics Committee of ORC Macro Inc. as well as Ethics Boards of the Haitian Bureau of Statistics and the Haitian Ministry of Public Health and Population. Since the data were not collected by the authors of this paper, permission was sought from MEASURE DHS website and access to the data was provided after our intent for the request was assessed and approved on May 3, 2022. Data are available on https://dhsprogram.com/data/available-datasets.cfm.

## Results

### Background Characteristic of the study population

Summary statistics of the analytic sample are shown in Table 1. Of the 6303 registered births, slightly more than 45% (45.9%) were to mothers aged 25–34 and 26.9% were to mothers aged 35 and above. The mean age of the mothers was 29.9 (SD ± 7.1) and the vast majority of them were Christians. About 65% of these children lived in rural areas, 18% came from the "Aire Métropolitaine de Port-au-Prince" region, and 18.9% were from the "Reste-Ouest". Slightly more than 45% of them were in the highest (poorest/poorer) wealth index quintiles, around 60% had mothers with a primary or no formal education, and a third had mothers with no income-generating activities. Almost all the mothers interviewed were not covered by health insurance. Furthermore, over than half of the children (53.4%) had mothers with less than 3 children. Less than 40% (*n* = 2237) of the total 6303 births were institutional (31.2 and 4.3% delivered at public and private sector health facility, respectively), and 52.8% of the children had an average birth weight. For 47.1% of them, their mothers had received 4–8 antenatal visits during pregnancy.

**Table 1:** Socio-demographic profile of the population study

Socio-demographic characteristics	*N*	Percentage
Mother's age		
Less than 25 years	1712	27.2
25–34	2895	45.9
35 and above	1696	26.9
Place of residence		
Urban	2209	35.1
Rural	4094	64.9
Region		
Aire Métropolitaine de Port-au-Prince	1137	18.0
Reste-Ouest	1194	18.9
Sud-Est	336	5.3
Nord	687	10.9
Nord-Est	243	3.9
Artibonite	988	15.7
Centre	542	8.6
Sud	411	6.5
Grand'Anse/Nippes	442	7.0
Nord-Ouest	322	5.1
Wealth Index		
Poorest	1582	25.1
Poorer	1303	20.7
Middle	1320	20.9
Richer	1203	19.1
Richest	895	14.2
Currently working		
Yes	4205	66.7
No	2098	33.3
Religion		
Christian	5525	87.7
Non-Christian	778	12.3
Mother's education level		
Primary or no formal education	3745	59.4
Secondary	2340	37.1
Higher	219	3.5
Covered by health insurance		
Yes	132	2.1
No	6171	97.9
Number of living children		
Less than 3	3369	53.4
3–4	1721	27.3
5 and above	1213	19.3
Number of antenatal visits		
Less than 4	1628	25.8
4–8	2971	47.1
9 and above	286	4.5
Don't know/missing	1418	22.5
Type of facility		
Public	1964	31.2
Private	273	4.3
Home/others	4066	64.5
Baby’s birth weight		
Average	3331	52.8
Low birth weight	1619	25.7
High birth weight	1353	21.5
Total	6303	100.0

### Prevalence of CS by selected socio-demographic characteristics

Table 2 includes information on CS by selected socio-demographic variables. The overall prevalence of CS was estimated at 5.4% (95% CI 4.8–6.0) in Haiti. The results showed regional disparities. The prevalence of CS was 9.7% in urban areas, while it was 3.1% in rural areas. Similarly, CS was most common in the "Aire Métropolitaine de Port-au-Prince" region (11.4%), and least common in the "Nord-Ouest" (2.5%). CS delivery was most prevalent among women aged 25–34 years (6.2%), and least prevalent among those under 25 years (4.6%). Women from richest households (18.1%), with a higher education level (28.6%), who received 9 or more antenatal visits during pregnancy (20%), who delivered in a private sector health facility (27.2%), and covered by health insurance (34.4%) exhibited the highest prevalences of CS. There was no significant difference between the prevalence of CS among women who had an income-generating activity (5.3%) and those who had no income-generating activity (5.5%). Furthermore, we found that CS use was much higher among Christian women (5.8%) than among non-Christian women (2.5%). Likewise, CS was most common among women who had less than 3 children (8.2%), and least common among those who had 5 children and above (1.2%). Children with high birth weight (big baby) (6.5%) were most frequent to be delivered through CS than those who had a low birth weight (5.9%) or an average birth weight (4.9%). All selected covariates had significant associations with CS (*p* < 0.05), except "currently working".

**Table 2:** Prevalence of CS delivery by socio-demographic characteristics

Socio-demographic characteristics^a^	Caesarean section		*p* value
	Yes (*N*/%)	No (*N*/%)	
Mother's age			0.037
Less than 25 years	78 (4.6)	1628 (95.4)	
25–34	178 (6.2)	2695 (95.8)	
35 and above	83 (4.9)	1606 (95.1)	
Place of residence			0.000
Urban	212 (9.7)	1969 (90.3)	
Rural	128 (3.1)	3961 (96.9)	
Region			0.000
Aire Métropolitaine de Port-au-Prince	127 (11.4)	989 (88.6)	
Reste-Ouest	48 (4.0)	1143 (96.0)	
Sud-Est	11 (3.3)	325 (96.7)	
Nord	38 (5.5)	648 (94.5)	
Nord-Est	13 (5.3)	230 (94.7)	
Artibonite	29 (2.9)	956 (97.1)	
Centre	29 (5.4)	513 (94.6)	
Sud	22 (5.4)	388 (94.6)	
Grand'Anse/Nippes	14 (3.2)	429 (96.8)	
Nord-Ouest	8 (2.5)	309 (97.5)	
Wealth Index			0.000
Poorest	20 (1.3)	1562 (98.7)	
Poorer	44 (3.4)	1257 (96.6)	
Middle	43 (3.3)	1268 (96.7)	
Richer	72 (6.0)	1119 (94.0)	
Richest	160 (18.1)	723 (81.9)	
Currently working			0.757
Yes	224 (5.3)	3966 (94.7)	
No	115 (5.5)	1963 (94.5)	
Religion			0.000
Christian	320 (5.8)	5174 (94.2)	
Non-Christian	19 (2.5)	755 (97.5)	
Mother's education level			0.000
Primary or no formal education	72 (1.9)	3664 (98.1)	
Secondary	206 (8.9)	2110 (91.1)	
Higher	62 (28.6)	155 (71.4)	
Covered by health insurance			0.000
Yes	44 (34.4)	84 (65.6)	
No	295 (4.8)	5845 (95.2)	
Number of living children			0.000
Less than 3	274 (8.2)	3072 (91.8)	
3–4	52 (3.0)	1663 (97.0)	
5 and above	14 (1.2)	1194 (98.8)	
Number of antenatal visits			0.000
Less than 4	35 (2.2)	1579 (97.8)	
4–8	201 (6.8)	2758 (93.2)	
9 and above	57 (20.0)	228 (80.0)	
Don't know/missing	45 (3.2)	1364 (96.8)	
Type of facility			0.000
Public	213 (10.9)	1735 (89.1)	
Private	70 (27.2)	187 (72.8)	
Home/others	55 (1.4)	4007 (98.6)	
Baby’s birth weight			0.000
Average	157 (4.7)	3157 (95.3)	
Low birth weight	95 (5.9)	1511 (94.1)	
High birth weight	87 (6.5)	1261 (93.5)	
Total	339 (5.4)	5929 (94.6)	

^a^Missing data (*n* = 35)

### Individual and community-level factors associated with CS in Haiti

The results from the final model indicated that the mother’s age, mother’s education level, being covered by health insurance, number of living children, number of antenatal visits, type of facility, and baby’s birth weight were significantly associated with CS (Table 3).

**Table 3:** Multilevel logistic regression estimates for CS by selected socio-demographic characteristics

Sociodemographic characteristics	Model-0 ICC = 31.6%	Model-I aOR (95% CI)	Model-II aOR (95% CI)	Model-III aOR (95% CI)
Mother’s age				
Less than 25 years		0.81 (0.59–1.11)		0.80 (0.58–1.11)
35 and above		1.40 (1.00–1.96)*		1.38 (1.00–1.96)*
Ref. = 25–34				
Wealth Index				
Richest		4.36 (2.39–7.94)***		1.92 (0.95–3.86)
Richer		2.28 (1.26–4.10)**		1.21 (0.61–2.38)
Middle		1.64 (0.90–2.98)		1.09 (0.57–2.06)
Poorer		2.22 (1.25–3.94)**		1.98 (1.10–3.54)
Ref. = poorest				
Religion				
Non-Christian		0.64 (0.38–1.08)		0.68 (0.39–1.16)
Ref. = Christian				
Mother's education level				
Secondary		2.07 (1.48–2.90)***		1.95 (1.39–2.76)***
Higher		4.15 (2.49–6.90)***		3.25 (1.92 – 5.49)***
Ref. = primary or no formal education				
Covered by health insurance				
Yes		2.98 (1.85–4.80)***		2.57 (1.57–4.19)***
Ref. = No				
Number of living children				
Less than 3		4.44 (2.36–8.35)***		4.13 (2.18–7.85)***
3–4		2.10 (1.11–3.98)*		2.07 (1.09–3.94)*
Ref. = 5 and above				
Number of antenatal visits				
4–8		1.39 (1.04–1.87)*		1.33 (0.98–1.79)
9 and above		2.39 (1.52–3.76)***		2.21 (1.40–3.50)**
Ref. = less than 4				
Baby’s birth weight				
Average		0.66 (0.50–0.90)**		0.66 (0.48–0.91)*
Low birth weight		1.03 (0.74–1.45)		1.05 (0.74–1.48)
Ref. = high birth weight				
Place of residence				
Urban			1.80 (1.16–2.81)**	1.22 (0.75–2.00)
Ref. = rural				
Region				
Reste-ouest			0.65 (0.33–1.29)	0.82 (0.41–1.62)
Sud-Est			0.64 (0.27–1.55)	0.65 (0.27–1.59)
Nord			0.78 (0.41–1.49)	1.03 (0.54–1.95)
Nord-Est			0.71 (0.31–1.60)	1.07 (0.47–2.42)
Artibonite			0.40 (0.21–0.78)**	0.53 (0.28 – 1.03)
Centre			0.86 (0.42–1.75)	1.21 (0.59–2.48)
Sud			0.77 (0.36–1.64)	0.87 (0.40–1.87)
Grand'Anse/Nippes			0.55 (0.25–1.20)	0.71 (0.32–1.57)
Nord-Ouest			0.42 (0.17–1.06)	0.53 (0.21–1.32)
Ref. = Aire Métropolitaine de Port-au-Prince				
Community*Type of facility				
Highly private			3.17 (2.17–4.65)***	1.90 (1.25–2.85)**
Highly home/others			0.24 (0.18–0.33)***	0.36 (0.26–0.50)***
Ref. = highly public				

*p < 0.05, **p < 0.01, ***p < 0.001

Mothers aged 35 and above were found to have a greater odds (aOR = 1.38; 95% CI 1.00–1.96) of CS compared with those aged 25–34 years. The odds of CS were higher among women who attended secondary (aOR = 1.95; 95% CI 1.39–2.76) and higher education level (aOR = 3.25; 95% CI 1.92–5.49) compared to those with primary level or no formal education. Similarly, mothers who were covered by health insurance were 2.6 times more likely (aOR = 2.57; 95% CI 1.57–4.19) to deliver children by CS as compared to those who were not covered by health insurance. Women with less than 3 children (aOR = 4.13; 95% CI 2.18–7.85) and those with 3–4 children (aOR = 2.07; 95% CI 1.09–3.94) were found to have a higher probability of delivering through CS. Likewise, the odds of CS among women who received 9 or more antenatal visits were 2.2 times higher (aOR = 2.21; 95% CI 1.40–3.50) than that of women who received less than 4 antenatal visits. Furthermore, children in communities with high preponderance of private health facilities had 1.9 greater odds (aOR = 1.90; 95% CI 1.25–2.85) to be delivered through CS. Finally, the odds of CS among children with an average birth weight (aOR = 0.66; 95% CI 0.48–0.91) was decreased by 34% than that of children with high birth weight.

### Measures of variation

In the null model (Model-0), there were substantial variations in CS across clusters (variance = 1.52; 95% CI 1.07–2.16). The null model also indicated that 31.6% of the total variance in CS practice was attributed to between-cluster variation. Besides, the PCV in the final model (Model-III) revealed that 48% of the variability in CS was explained by both individual and community-level characteristics (Table 4).

**Table 4:** Measure of variation for CS in Haiti, HDHS 2016–2017

Measure of variation	Model-0	Model-I	Model-II	Model-III
Variance	1.52 (1.07–2.16)	0.73 (0.46–1.17)	0.91 (0.60–1.37)	0.79 (0.50–1.26)
ICC (%)	31.58	18.26	21.63	19.40
PCV (%)	Reference	51.97	40.13	48.03
Model fitness				
Log-likelihood	− 1241.05	− 1065.40	− 1115.13	− 1025.16
AIC	2486.11	2166.80	2258.26	2110.33

*ICC* intra-class Correlation Coefficient, *PCV* proportional change in variance, *AIC* Akaike Information Criterion

## Discussion

This study aimed to determine individual and community-level factors associated with CS in Haiti. We found that the prevalence of CS was 5.4% (95% CI 4.8–6.0) in Haiti. Results also revealed that mother’s age, mother’s education level, being covered by health insurance, number of living children, number of antenatal visits, type of facility, and baby’s birth weight were significantly associated with CS.

Greater maternal age is an important predictor associated with CS in Haiti. Supported by studies conducted in Europe [61], Asia [8, 62], Oceania [63], and Africa [24, 26], our findings indicated that women aged 35 years and above were more likely to undergo CS than those aged 25–34 years. The reasons for the increased likelihood of CS among women aged 35 years and above remain unclear [8, 64, 65]; however, it might be due to the fact that women of advanced maternal age (specifically first-time mothers) are more likely to have hypertension, diabetes, and experience preterm delivery [66], important risks factors for CS [8].

Women with higher education levels had increased odds of CS delivery, which reflects findings from past studies [24, 67, 68]. Arguably, educated women have better access to obstetric care services and information about maternal and child health including CS [57, 69]. Furthermore, our data indicated that women with higher education levels were more likely to give births at advanced maternal ages. As discussed earlier, the probability of delivery by CS increases with rising age of the mothers [8].

It was found that being covered by health insurance was associated with higher CS Birth rates. Mothers who were covered by health insurance were more likely to have a CS birth compared to those who were not covered. This evidence is consistent with other studies [37, 38]. In Haiti, CS is a high-cost procedure, especially in private health facilities [70]. When women are covered by health insurance, it allows them to partially or totally cover the fees related to this surgical procedure [6, 70].

Furthermore, women who received 9 or more antenatal visits during pregnancy were more likely to have CS compared to those who received less than 4 antenatal visits. This observation is in agreement with other reports from different low-income countries [29, 33]. The more antenatal visits women receive, the more interaction they have with healthcare professionals, which might influence them to deliver through CS [37, 53]. Moreover, we cannot exclude the fact that women who received 9 or more antenatal visits might be women who had pregnancy complications, hence their preference for CS delivery [8, 27].

The likelihood of CS was higher in communities with high preponderance of private health facilities. This evidence corroborates the association found in past studies [36, 71]. Public health facilities in Haiti are poorly financed, staffed and equipped [72, 73]. Therefore, if a woman's delivery requires a CS, she will be more likely (if she has the financial means) to opt for a private health facility. Several researchers also argued that in developing countries, including Haiti, the considerable increase of the CS rate in private health facilities could be due to the financial benefits [54, 74], which would lead to an increase in the rate of unnecessary CS deliveries [8]. It should also be noted that in many rural and remote areas in Haiti, there are no public health facilities [73]. To ensure that women with high-risk pregnancies have safe deliveries in these communities characterized by generalized poverty, government authorities need to subsidize CS and improve access to obstetric care services.

Children with big size at birth had higher odds of CS compared to those with an average size. Former studies [6, 55] affirmed this association between size of child at birth and CS. Big babies often have difficulties during deliveries (birth complications) due to insufficient passage and prolonged labor [6, 56]. As reported in many studies [75, 76], insufficient passage and prolonged labor are common causes of CS.

Finally, we found that the more children women had, the less likely they were to undergo CS delivery. Our results align with other studies conducted in Rwanda [6], Kenya [39], and Ethiopia [24, 77]. A possible explanation is that women who had 5 or more children had previously given birth successfully or experienced fewer complications requiring CS [24, 69, 78]. In addition, it should be noted that our data showed that women who had fewer children were those with higher education levels and gave births more at advanced maternal ages. Socio-demographic factors such as education level and mother's age were reported as predictors of CS in Haiti.

### Study strengths and limitations

The key strength of this study lies in the use of the 2016–2017 HDHS (a nationally representative data) and a large sample size (*n* = 6303 children born in 5 years prior the survey). This enables the generalizability of the findings of CS in Haiti. In addition, the findings are based on adequate statistical power (data were weighted for the sampling probabilities) and took into account the complex sampling procedures in the analysis. However, the study has some limitations. First, due to the cross-sectional study design, we could not infer causality in the relationships between the covariates and the outcome variable. Second, the study was limited by the use of secondary data restricting study variables. Third, due to the self-reported nature of the DHS surveys, the data may be subject to recall bias.

## Conclusion

Based on the findings of this study, the overall prevalence of CS in Haiti was low (5.4%). Using the 2016–2017 HDHS, it also showed that mother’s age, mother’s education level, being covered by health insurance, number of living children, number of antenatal visits, type of facility, size of child at birth were significant predictors of CS deliveries. In summary, the results of this study showed deep community and socioeconomic disparities in the use of CS in Haiti. To ensure that women with high-risk pregnancies have safe deliveries in rural and remote areas characterized by generalized poverty, government authorities need to subsidize CS, increase access to this surgical intervention, recruit skilled birth attendants (SBA), and improve access to obstetric care services. However, note that some doctors in private health facilities in Haiti forced women to opt for CS (for their own convenience, quick handling to save time or economic incentives), regardless of the fact that they could deliver the baby naturally. To make informed choices about their delivery, women need to receive adequate information about the risks and benefits of CS. A monitoring system must be established by the Ministry of Public Health and Population to drastically reduce the overuse of CS and promote vaginal deliveries whenever women are not at hight risk. Mothers and newborns are at risk of unnecessary deaths and complications due to underuse or unavailability of CS services at the healthcare facilities, while overuse or performing CS when not required may cause unnecessary complications to be experienced by mothers and newborns.

## Data Availability

The data used in this study is publicly available at: https://dhsprogram.com/data/availabledatasets.cfm.
